# Association between Thyroid Function and Ocular Parameters

**DOI:** 10.3390/biology11121847

**Published:** 2022-12-18

**Authors:** Mirjana Babić Leko, Nikolina Pleić, Mladen Lešin, Ivana Gunjača, Vesela Torlak, Jelena Škunca Herman, Zoran Vatavuk, Ante Punda, Ozren Polašek, Caroline Hayward, Tatijana Zemunik

**Affiliations:** 1 Department of Medical Biology, School of Medicine, University of Split, 21000 Split, Croatia; 2 Department of Ophthalmology, University Hospital of Split, 21000 Split, Croatia; 3 Department of Nuclear Medicine, University Hospital of Split, 21000 Split, Croatia; 4 Department of Ophthalmology, Clinical Hospital Sisters of Mercy, 10000 Zagreb, Croatia; 5 Department of Public Health, School of Medicine, University of Split, Šoltanska 2, 21000 Split, Croatia; 6 Algebra University College, 10000 Zagreb, Croatia; 7 MRC Human Genetics Unit, Institute of Genetics and Cancer, University of Edinburgh, Edinburgh EH4 2XU, UK

**Keywords:** ocular parameters, thyroid hormones, TSH, hypothyroidism, hyperthyroidism, corneal thickness, intraocular lens power, anterior chamber angle, spherical power

## Abstract

**Simple Summary:**

The scope of this study was to determine if various ocular parameters are associated with parameters indicating thyroid activity. It is well-known that in some pathological thyroid conditions, the eyes can also be affected. Thyroid eye disease is the most extreme example of eye pathology caused by a pathological thyroid condition. In the current study, we measured the plasma levels of thyroid-stimulating hormone (TSH), free triiodothyronine (fT3), free thyroxine (fT4), thyroglobulin (Tg), thyroglobulin antibodies (TgAb), and thyroid peroxidase antibodies (TPOAb), in addition to 20 ocular parameters in 4633 healthy adults (10 for each eye, including corneal radius, corneal thickness, anterior chamber depth, anterior chamber angle, lens thickness, posterior chamber length, axial length, intraocular lens power (IOL), spherical power, and cylinder power). This is, to the best of our knowledge, the largest study inferring the effect of thyroid activity on ocular parameters. The most important result of this study was that patients with hyperthyroidism had thicker corneas compared to euthyroid individuals. Intraocular lens power was increased in patients with clinical hypothyroidism, while spherical power was increased in euthyroid individuals with positive antibodies. The diagnostic potential of corneal thickness monitoring in patients with hyperthyroidism, which could indicate the later development of thyroid-related eye disease, should be explored in longitudinal studies.

**Abstract:**

During development, thyroid hormones play an important role in eye development, while in adults, some pathological thyroid conditions can affect the normal functioning of the eyes. Thyroid eye disease is the most well-known eye pathology caused by a pathological thyroid condition. Few studies have investigated the association between ocular parameters and thyroid function. Thus, in this study, we aimed to examine whether thyroid activity affects ocular parameters. This cross-sectional study included 4633 healthy adults recruited within the 10,001 Dalmatians project of the Croatian Biobank. The plasma levels of thyroid-stimulating hormone (TSH), free triiodothyronine (fT3), free thyroxine (fT4), thyroglobulin (Tg), thyroglobulin antibodies (TgAb), and thyroid peroxidase antibodies (TPOAb) were measured by an immunoassay. We determined 20 ocular parameters for each participant (10 for each eye, including corneal radius, corneal thickness, anterior chamber depth, anterior chamber angle, lens thickness, posterior chamber length, axial length, intraocular lens power (IOL), spherical power, and cylinder power). Patients with hyperthyroidism had thicker corneas compared to euthyroid individuals. Corneal thickness was also negatively associated with plasma TSH levels. Intra-ocular lens power was higher in patients with clinical hypothyroidism, while spherical power was higher in euthyroid individuals with positive antibodies compared to euthyroid individuals. Intra-ocular lens power negatively correlated with fT4 levels, while spherical power positively correlated with TgAb, TPOAb, and Tg levels and negatively correlated with TSH levels. The anterior chamber angle was positively associated with plasma TSH levels and TPOAb levels and negatively associated with plasma fT4 levels. These findings suggest an interesting interplay between ophthalmic measures and thyroid status, detectable even in the general adult population.

## 1. Introduction

Thyroid hormones (triiodothyronine (T3) and thyroxine (T4)) are crucial during neuronal development [1,2]. Since parts of the eye, such as the retina, iris, optic nerves, and ciliary body, arise from the neuroepithelium [3], thyroid hormones play an important role in eye development. In particular, thyroid hormones play a crucial role in the normal development of the retina—more precisely, the retinal cone photoreceptors (crucial for colour vision) [4].

In adults, some pathological thyroid conditions can affect the normal functioning of the eyes. The most extreme example of an eye pathology caused by a pathological thyroid condition is thyroid eye disease (TED), also called Graves’ eye disease, Graves’ orbitopathy, or Graves’ ophthalmopathy. This condition, caused by a hyperactive thyroid, is characterised by attacks on the eye tissues by the immune system that lead to the expansion of the eye muscles or fat [5]. It has been speculated that hypothyroidism could contribute to the development of glaucoma [6]. However, insufficient evidence supports this hypothesis [7]. Smith et al. hypothesised that hypothyroidism could contribute to the increased accumulation of mucopolysaccharides in the trabecular meshwork (the tissue area in the eye responsible for the drainage of aqueous humour from the ciliary body into the anterior chamber). This results in decreased aqueous drainage that causes increased intraocular pressure (IOP), leading to glaucoma [8]. Some studies also detected an increased risk of dry eye disease in both hypothyroidism [9] and hyperthyroidism [10].

Despite the substantial evidence of altered thyroid function contributing to the development of eye pathology, only a few studies (mainly including an insufficient number of participants) investigating the association between ocular parameters and thyroid function have been conducted so far. Thus, the scope of this study was to test if thyroid activity affects ocular parameters. To this end, we measured ocular biometry and refraction in a large cohort of 4633 participants and compared them to six indicators of thyroid activity (thyroid-stimulating hormone (TSH), free T3 (fT3), free T4 (fT4), thyroglobulin (Tg), thyroglobulin antibodies (TgAb), and thyroid peroxidase antibodies (TPOAb)). We also compared the levels of measured ocular parameters between euthyroid individuals and individuals with altered thyroid function.

## 2. Materials and Methods

### 2.1. Participants

This cross-sectional study included 4848 participants over 18 years of age. Participants were recruited within the 10,001 Dalmatians project, which is part of the Croatian Biobank program [11]. All participants were from the Dalmatian region of South Croatia (1025 from the island of Vis, 2811 from the island of Korčula, and 1012 from the city of Split). Participants who underwent thyroid surgery or were taking thyroid medication were excluded from the study, and measurements from eyes with a history of trauma, intraocular surgery, or LASIK eye surgery were removed (215 patients); thus, a total of 4633 participants were included in the analysis (2805 (60.5%) women and 1828 (39.5%) men). Women who were pregnant or suspected to be pregnant were not included in our study. Demographic data and a summary of measured variables of all included participants are presented in Table 1. Written informed consent was obtained from participants. All procedures were approved by the Ethical board of the University of Split School of Medicine (No: 2181-198-03-04-14-0031 and 2181-198-03-04-19-0022).

### 2.2. Biochemical Measurements

After an overnight fast, blood samples were obtained in the morning between 7 a.m. and 9 a.m. Plasma TSH, fT3, fT4, Tg, TgAb, and TPOAb levels were measured by an immunoassay using a fully automated Biomedica Liaison Chemiluminescence Analyzer (DiaSorin, Saluggia, Italy). The reference ranges for our population were: TSH, 0.3 to 3.6 mIU/L; fT3, 3.39 to 6.47 pmol/L; fT4, 10.29 to 21.88 pmol/L; Tg, 0.2–50 ng/mL; TgAb, 5–100 IU/mL; and TPOAb levels, 1–16 IU/mL. Biochemical analyses were carried out in the Laboratory of Biochemistry, Department of Nuclear Medicine, University Hospital Split.

### 2.3. Measurement of Ocular Parameters

Keratometry (CC) and noncycloplegic autorefraction were measured on each eye with a hand-held autorefractometer/keratometer (Ark30; Nidek, Gamagori, Japan). Refraction was analysed as the spherical and cylinder power. CC was the average of the values of corneal radii of curvature from the two principal meridians. In addition, we measured three readouts intended for the intraocular lens power (IOL), which correlated to the lens capsule contour and were considered an additional ophthalmic phenotype. Central corneal thickness was recorded along with other ocular biometric measurements using a Nidek Echoscan US-1800 A-scan after applying sterile oxybuprocaine anaesthetic eye drops (Minims-Chauvin Pharmaceuticals Ltd., Surrey, UK).

### 2.4. Definitions

Euthyroid individuals are those with TSH, fT3, and fT4 levels within the reference ranges, without the presence of TgAb and TPOAb. Euthyroid individuals with positive antibodies are those that have TPOAb and/or TgAb above reference ranges but TSH, fT3, and fT4 levels within the reference ranges. Individuals with subclinical hypothyroidism have TSH levels above 3.6 mlU/L and fT4 and fT3 within the reference ranges, while individuals with clinical hypothyroidism have TSH > 3.6 mlU/L, fT4 < 10.29 pmol/L, and fT3 ≤ 6.47 pmol/L. Individuals with subclinical hyperthyroidism have TSH levels below 0.3 mlU/L and fT4 and fT3 within the reference ranges, while individuals with clinical hyperthyroidism have TSH < 0.3 mlU/L, fT4 > 21.88 pmol/L, and fT3 ≥ 3.39 pmol/L.

### 2.5. Statistical Analyses

Ocular parameters were compared between individuals with euthyroidism and individuals with altered thyroid function using an independent samples *t*-test. The association of ocular parameters with TSH, thyroid hormones, Tg, TgAb, and TPOAb levels was tested by correlation (using Pearson’s and Spearman’s correlation coefficient) and by linear regression (Supplementary Table S1). An unsupervised machine learning method, principal component analysis (PCA), was used to reduce the dimensionality of ocular parameters into fewer factors, i.e., groups. Bartlett’s χ^2^ test of sphericity and the Kaiser–Meyer–Olkin index were used to assess the adequacy of test items and sample size for factor analysis. PCA reduced the list of 20 ocular parameters to key groups (factors). Orthogonal (varimax) rotation was used in factor analysis to extract factors that were independent or uncorrelated with each other. When determining the number of factors, we considered the interpretability of the results, the visual inspection of a scree plot, and eigenvalues greater than 1. If the absolute factor loading value was ≥0.4 for an ocular parameter item, it was considered that the item loaded on a given factor (factor loading is a measure of the correlation between an item and a factor). Finally, PCA provided factor-specific scores for each participant (calculated using the regression method). The association of the PCA-obtained ocular parameter groups with TSH, thyroid hormones, Tg, TgAb, and TPOAb levels was carried out using multiple linear regression analyses. Ocular parameter groups were considered dependent variables, while hormone levels were considered independent variables. The linear regression models were controlled for gender and age. Before regression analysis, we tested regression assumptions (normality of residuals, linearity of the data, and homoscedasticity) using diagnostic plots. *p* values < 0.05 were considered statistically significant. Statistical analyses were performed using R (R Foundation for Statistical Computing, Vienna, Austria).

## 3. Results

### 3.1. Comparison of Ocular Parameters between Individuals with Euthyroidism and Individuals with Altered Thyroid Function

**Figure 1:**
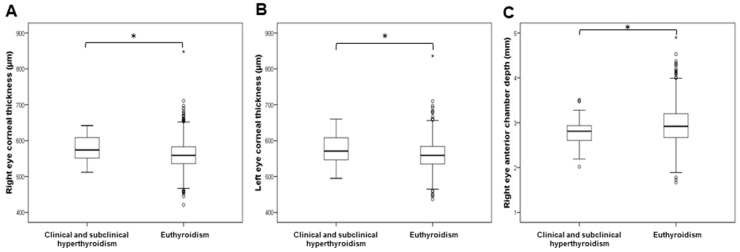
Comparison of ocular parameters between individuals with clinical or subclinical hyperthyroidism and euthyroid individuals. (**A**) Right eye corneal thickness, (**B**) left eye corneal thickness, (**C**) right eye anterior chamber depth. * *p* < 0.05.

**Figure 2:**
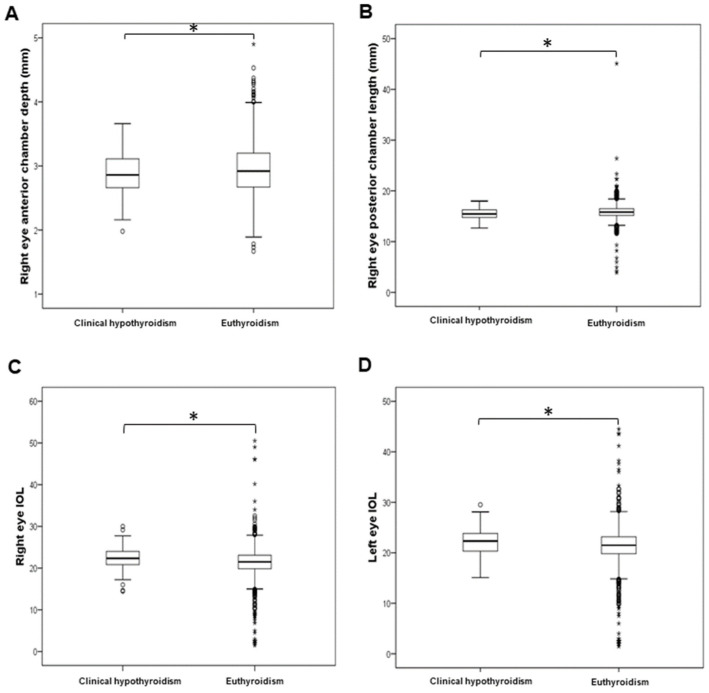
Comparison of ocular parameters between individuals with clinical hypothyroidism and euthyroid individuals. (**A**) Right eye anterior chamber depth, (**B**) right eye posterior chamber length, (**C**) right eye IOL, (**D**) left eye IOL. * *p* < 0.05.

**Figure 3:**
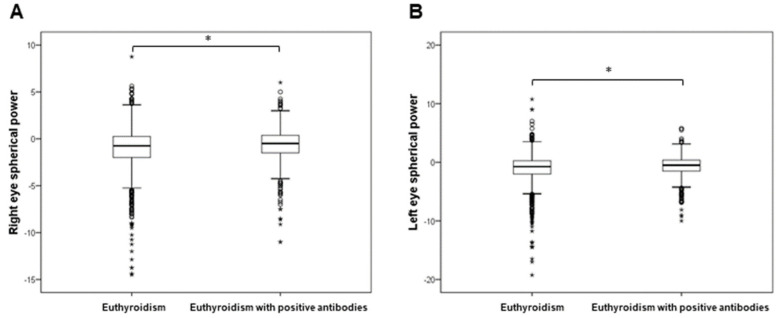
Comparison of ocular parameters between euthyroid individuals with positive antibodies and euthyroid individuals. (**A**) Right eye spherical power, (**B**) left eye spherical power. * *p* < 0.05.

The levels of ocular parameters were compared between 2514 individuals with euthyroidism and groups of patients with subclinical hypothyroidism (275), clinical hypothyroidism (107), subclinical and clinical hyperthyroidism (40), and euthyroidism with positive antibodies (656). The analysed optical parameters, as well as demographic data and biochemical measurements, across the thyroid function groups are presented in Supplementary Table S2. Since we only included four participants with clinical hyperthyroidism, we combined participants with subclinical and clinical hyperthyroidism into a single group. The corneal thickness of both the right (t = 2.969; df = 2541; *p* = 0.003) and left eye (t = 2.572; df = 2533; *p* = 0.010) was significantly increased in patients with subclinical and clinical hyperthyroidism in comparison to euthyroid individuals (Figure 1). The anterior chamber depth of the right eye was significantly decreased in patients with subclinical and clinical hyperthyroidism compared to euthyroid individuals (t = −2.403; df = 2551; *p* = 0.016; Figure 1). The anterior chamber depth (t = −1.999; df = 2619; *p* = 0.046) and posterior chamber length (t = −2.277; df = 2619; *p* = 0.023) of the right eye were significantly decreased in patients with clinical hypothyroidism in comparison to euthyroid individuals (Figure 2). The IOL levels of both the right (t = 2.795; df = 2587; *p* = 0.005) and left eye (t = 2.704; df = 2587; *p* = 0.007) were significantly increased in patients with clinical hypothyroidism in comparison to euthyroid individuals (Figure 2). The spherical power of both the right (t = 3.699; df = 1103.2; *p* < 0.001) and left eye (t = 3.274; df = 1202.8; *p* = 0.001) was significantly increased in euthyroid individuals with positive antibodies in comparison to euthyroid individuals (Figure 3).

### 3.2. Principal Component Analysis of Ocular Parameters

This study included 20 numeric ocular parameters. However, two variables were excluded from the PCA. The variables of right and left eye corneal radius were excluded from the PCA analysis as these variables were determined in a lower number of participants (2257 and 2272, respectively) in comparison to the other variables (determined in a higher number of participants; Table 1). Thus, factor analysis included 18 ocular parameters that were classified into 7 groups (factors) upon analysis. These factors explained 72.36% of the total variance in the analysed ocular parameters. Bartlett’s test of sphericity (*p* < 0.001) and the Kaiser–Meyer–Olkin measure of sampling adequacy (0.732) supported the suitability of data for PCA. The obtained factors with corresponding ocular parameters and their loading values are provided in Table 2. PCA successfully grouped measurements of the same parameter in the left and right eye into a single group (factor). Factor 1 captured the majority of the total variance and included the posterior chamber and axial length and the IOL of the eyes. Factor 2 captured the corneal thickness, Factor 3 the lens thickness, Factor 4 the cylinder power, Factor 5 the spherical power, Factor 6 the anterior chamber depth, and Factor 7 the anterior chamber angle. This dimensionality reduction enabled us to use the resulting factors as dependent variables to model the effect of thyroid hormones on the eye.

### 3.3. The Effect of TSH, Thyroid Hormones, Tg, TgAb, and TPOAb on the Eye

All analyses were controlled for the confounding effect of age and gender. Before analysis, TSH levels were log-transformed (since TSH distribution was right-skewed, following an approximately log-normal distribution). Linear regression analysis showed that plasma TSH levels were a negative predictor (β = −0.043, SE = 0.025, *p* = 0.016) of corneal thickness (factor 2). Plasma TSH levels, as well as TPOAb levels, were positive predictors (β_TSH_ = 0.042, SE = 0.025, *p* = 0.021, β_TPOAb_ = 0.038, SE = 0.000, *p* = 0.033), while fT4 was a negative predictor (β_fT4_ = −0.035, SE = 0.010, *p* = 0.05) of the anterior chamber angle (factor 7) (Table 3). Linear regression analysis showed no association between fT3 and TgAb levels and any of the analysed factors. Tg was a positive predictor (β_Tg_ = 0.050, SE = 0.001, *p* = 0.004) of lens thickness (Table 3). Simple correlation (without the correction for the confounding effect of age and sex) confirmed the associations above and further showed a negative correlation between Tg levels and Factor 1 (with high loadings for right and left eye posterior chamber length, right and left eye axial length, and right and left eye IOL; r_S_ = −0.054, *p* = 0.003) (Supplementary Table S1).

## 4. Discussion

The main scope of this study, which included 4633 participants, was to test if various ocular parameters (corneal radius, corneal thickness, anterior chamber depth, anterior chamber angle, lens thickness, posterior chamber length, axial length, intraocular lens power (IOL), spherical power, and cylinder power) differed between euthyroid individuals and individuals with altered thyroid function. Additionally, ocular parameters were correlated with TSH, thyroid hormones, Tg, TgAb, and TPOAb levels. The most significant result of this study was the presence of thicker corneas in patients with hyperthyroidism compared to euthyroid individuals. Correlation analysis also confirmed this finding (with the corneal thickness negatively associated with plasma TSH levels). A possible explanation for this observation is that patients with hyperthyroidism often develop ocular surface disorders, such as dry eyes [12]. Since it was shown that patients with dry eye disease have an increase in corneal epithelium thickness [13,14], corneal thickness in patients with hyperthyroidism should be monitored. We hypothesise that these corneal changes could be an indicator of early inflammatory changes associated with the later development of thyroid-related eye disease. Additionally, intraocular lens power (IOL) was significantly increased in patients with clinical hypothyroidism, and this result was further confirmed by correlation analysis (since IOL negatively correlated with fT4 levels). Spherical power was significantly increased in euthyroid individuals with positive antibodies compared to euthyroid individuals. Spherical power also correlated positively with TgAb, TPOAb, and Tg levels and negatively with TSH levels. Although the measure of the anterior chamber angle was positively associated with plasma TSH levels and TPOAb levels, and negatively associated with plasma fT4 levels, no significant difference in the measure of the anterior chamber angle was observed between euthyroid individuals and individuals with altered thyroid function. Anterior chamber depth was significantly decreased in both hypothyroidism and hyperthyroidism, while posterior chamber length was significantly decreased in clinical hypothyroidism.

Previous studies mostly analysed corneal thickness in patients with TED [15,16] and hypothyroidism [7,17,18,19,20,21]. In a study that included 27 individuals with TED and 30 healthy individuals, Karabulut et al. did not observe a significant difference in central corneal thickness between patients with TED and healthy individuals (with Goldmann-correlated intraocular pressure (IOPg) and corneal-compensated IOP (IOPcc) being significantly increased and corneal hysteresis (CH) being significantly decreased in patients with TED) [15]. Additionally, in a study that included 75 patients with TED and 57 healthy individuals, central corneal thickness did not differ significantly between the groups (with CH being significantly decreased in patients with TED) [16]. Other studies observed further ocular surface parameter alterations in patients with TED [22,23,24].

In a study that included 33 patients with primary overt hypothyroidism, Ozturk et al. observed no change in central corneal thickness due to hypothyroidism or therapy for hypothyroidism [7]. Furthermore, the authors did not observe a change in IOP, anterior chamber parameters, mean retinal thickness, or mean retinal nerve fibre layer (RNFL) in hypothyroidism [7]. Roszkowska et al. tested the association between congenital hypothyroidism and keratoconus (characterised by the thinning and bulging of the cornea in a cone shape) [20]. In their study that included 31 subjects with congenital hypothyroidism and 19 healthy individuals, they observed no change in corneal parameters (central corneal thickness, corneal curvature, corneal volume and anterior elevation, and posterior elevation at the thinnest point) in diagnosed and treated congenital hypothyroidism [20]. A study that included 40 patients with euthyroid Hashimoto’s thyroiditis (HT) and 48 healthy controls found no difference in central corneal thickness, retinal thickness, and IOP between these two groups, while the levels of TPOAb negatively correlated with the retinal thickness [25]. Additionally, a study that included 48 patients with HT and 49 healthy controls did not observe a significant difference in central corneal thickness between these two groups, while IOPcc was significantly higher in the HT group [17]. However, Bassiouny et al. concluded that thyroid gland dysfunction contributes to the development of corneal tomographic changes [21]. In a study that included 50 patients with thyroid gland dysfunction and 50 healthy individuals, the authors observed a significant increase in central corneal thickness levels in patients with hypothyroidism in comparison to both healthy individuals and patients with hyperthyroidism [21]. Additionally, central corneal thickness levels positively correlated with TSH levels and negatively correlated with T4 levels [21]. Finally, patients with non-autoimmune thyroid gland dysfunction had thinner corneas in comparison to patients with autoimmune thyroid gland dysfunction [21]. Bahceci et al. hypothesised that hypothyroidism causes a reversible increase in corneal thickness and IOP [19]. In their study, they observed a significant decrease in corneal thickness and IOP levels after the treatment for hypothyroidism. Additionally, in the ninth month of the therapy, Bahceci et al. observed a negative correlation between fT3 and fT4 levels and corneal thickness and a positive correlation between TSH levels and corneal thickness [19]. A significant decrease in central corneal thickness values was also observed in 27 patients with HT (in comparison to 35 healthy individuals) in the study by Okan Olcaysü et al. [18]. Additionally, this study showed a significant increase in IOP and a significant decrease in subfoveal choroidal thickness (SFCT) and RNFL in HT patients in comparison to healthy individuals [18]. Additionally, it is important to note that corneal thickness also changes in healthy adults during aging in the absence of corneal pathology. However, studies have provided conflicting results. Some research groups observed a positive correlation between central corneal thickness and age [26,27], while others [28,29,30] observed that the cornea became thinner with age. Additionally, in healthy women, corneal thickness changes during the menstrual cycle (with the maximum thickness observed during ovulation) [31]. Moreover, study that included pregnant women observed that the changes in corneal biomechanics and topography that occur during pregnancy could be caused by changes in thyroid hormones during the pregnancy [32].

Of the other ocular parameters analysed in our study, we found that only anterior chamber depth and IOL power have previously been associated with thyroid gland function. Anterior chamber depth was both altered [33,34] and normal [35] in patients with TED. Hypothyroidism did not affect anterior chamber parameters (anterior chamber volume, depth, and angle) [7]. A change in anterior chamber depth and volume was not observed in congenital hypothyroidism [20]. IOL power decreased in 64.3% of patients with Graves’ disease after treatment with methimazole, a thionamide antithyroid drug [36].

When comparing our results to the results of other studies, we noticed that the studies investigating corneal thickness in patients with TED did not observe a significant difference in corneal thickness compared to healthy individuals [15,16]. However, our study showed the presence of thicker corneas in patients with hyperthyroidism compared to healthy individuals. In patients with hypothyroidism, either no change [7,17,20,25], a decrease [18], or an increase [19,21] in corneal thickness compared to healthy individuals was observed. However, it should be noted that our study lacked ethnic diversity, since it included only Caucasians of Croatian origin. The results of our study support those studies that did not observe a change in corneal thickness in hypothyroidism [7,17,20,25]. A patient with hyperthyroidism had a shallow anterior chamber depth [34], which was in accordance with our results, since a decrease in anterior chamber depth was observed in patients with hyperthyroidism. Additionally, Ozturk et al. did not observe a change in anterior chamber depth in hypothyroidism [7], which conflicted with the results of our study, since we observed a decrease in anterior chamber depth in hypothyroidism.

The main strength of our study was its large sample size, representing 4633 participants. This is, to our knowledge, the biggest cohort in which the effect of thyroid activity on ocular parameters has been investigated. We measured a total of 20 ocular parameters (10 for each eye, including corneal radius, corneal thickness, anterior chamber depth, anterior chamber angle, lens thickness, posterior chamber length, axial length, intraocular lens power (IOL), spherical power, and cylinder power) and compared them to six indicators of thyroid activity (TSH, fT3, fT4, Tg, TgAb, and TPOAb) and between euthyroid individuals and individuals with altered thyroid function. A limitation of this study was that we did not measure IOP and some other ocular parameters (such as retinal thickness, RNFL, and other anterior chamber parameters). Additionally, we did not have information on the presence of thyroid eye disease or dry eye status in the studied individuals. Finally, only four patients who suffered from clinical hyperthyroidism were included in this study.

## 5. Conclusions

In conclusion, the most important result of this study (which is, to the best of our knowledge, the largest study to investigate the effect of thyroid activity on ocular parameters) was that patients with hyperthyroidism had thicker corneas compared to euthyroid individuals. Intraocular lens power was increased in patients with clinical hypothyroidism, while spherical power was increased in euthyroid individuals with positive antibodies. The association of the aforementioned ocular parameters with thyroid activity was also confirmed by correlation analysis (corneal thickness—negatively associated with plasma TSH levels; intra-ocular lens power—negatively correlated with fT4 levels; spherical power—positively correlated with TgAb, TPOAb, and Tg levels and negatively correlated with TSH levels). Finally, the measure of the anterior chamber angle was positively associated with plasma TSH levels and TPOAb levels and negatively associated with plasma fT4 levels. In conclusion, the diagnostic potential of corneal thickness monitoring in patients with hyperthyroidism, which could indicate the later development of thyroid-related eye disease, should be explored in longitudinal studies.

## Figures and Tables

**Table 1:** Clinical characteristics of study participants.

Variable	Mean ± SD or Median (25–75th Percentile)	Number of Participants
Age	55 (42–66)	4578
TSH (mIU/L)	1.6 (1.1–2.5)	4420
fT4 (pmol/L)	12.9 (11.9–14.1)	4424
fT3 (pmol/L)	4.4 (4.2–4.8)	4395
Tg (ng/mL)	10.1 (5.5–16.8)	3491
TgAb (IU/mL)	8.5 (5–19.2)	4427
TPOAb (IU/mL)	4.4 (1.7–11.6)	4426
Right eye: posterior chamber length (mm)	15.8 (15.2–16.5)	3931
Right eye: axial length (mm)	23.1 ± 1.3	3933
Right eye: IOL (D)	21.5 (19.8–23.1)	3887
Right eye: corneal thickness (µm)	560.1 ± 36.2	3919
Right eye: lens thickness (mm)	4.4 ± 0.5	3903
Right eye: cylinder power (°)	−0.284 ± 0.922	3910
Right eye: spherical power (D)	−0.620 (−1.750–0.250)	3902
Right eye: anterior chamber angle (°)	88.5 ± 57.1	3331
Right eye: anterior chamber depth (mm)	2.9 ± 0.4	3933
Right eye: corneal radius (mm)	7.8 ± 0.3	2257
Left eye: posterior chamber length (mm)	15.9 ± 1.2	3921
Left eye: axial length (mm)	23.1 (22.4–23.7)	3929
Left eye: IOL (D)	21.6 (19.8–23.2)	3891
Left eye: corneal thickness (µm)	560.4 ± 36.2	3908
Left eye: lens thickness (mm)	4.4 ± 0.4	3904
Left eye: cylinder power (°)	−0.294 ± 1.291	3916
Left eye: spherical power (D)	−0.620 (−1.870–0.250)	3916
Left eye: anterior chamber angle (°)	89.1 ± 58.6	3329
Left eye: anterior chamber depth (mm)	2.9 ± 0.4	3929
Left eye: corneal radius (mm)	7.8 (7.6–8)	2272

fT3, free triiodothyronine; fT4, free thyroxine; IOL, intraocular lens power; Tg, thyroglobulin; TgAb, thyroglobulin antibodies; TPOAb, thyroid peroxidase antibodies; TSH, thyroid-stimulating hormone.

**Table 2:** List of ocular parameters and their factor loadings for seven groups (factors) identified by the principal component analysis.

Factors	Ocular Parameters (Factor Loadings)
Factor 1	Right eye: posterior chamber length (0.636), right eye: axial length (0.736), right eye: IOL (−0.746), left eye: posterior chamber length (0.710), left eye: axial length (0.715), left eye: IOL (−0.744)
Factor 2	Right eye: corneal thickness (0.939), left eye: corneal thickness (0.944)
Factor 3	Right eye: lens thickness (0.712), left eye: lens thickness (0.695)
Factor 4	Right eye: cylinder power (0.702), left eye: cylinder power (0.654)
Factor 5	Right eye: spherical power (0.542), left eye: spherical power (0.578)
Factor 6	Right eye: anterior chamber depth (0.512), left eye: anterior chamber depth (0.575)
Factor 7	Right eye: anterior chamber angle (0.672), left eye: anterior chamber angle (0.726)

IOL, intraocular lens power.

**Table 3:** Statistically significant associations of TSH, fT4, Tg, and TPOAb with ocular parameter groups (factors).

	Factor 2—Corneal Thickness	Factor 3—Lens Thickness	Factor 7—Anterior Chamber Angle
TSH	β = −0.043 *		β = 0.042 *
fT4			β = −0.035 *
Tg		β = 0.050 **	
TPOAb			β = 0.038 *

Effect size estimates of the associations as evaluated by regression analyses adjusted for age and gender. * *p* < 0.05, ** *p* < 0.01.

## Data Availability

Not applicable.

## Supplementary Materials

The following supporting information can be downloaded at: https://www.mdpi.com/article/10.3390/biology11121847/s1, Table S1: Correlation of optical parameters with TSH, thyroid hormones, Tg, TgAb, and TPOAb levels, Table S2: Analysed optical parameters, as well as demographic data and biochemical measurements across the thyroid function groups.

## Abbreviations

CC, keratometry; CH, corneal hysteresis; fT3, free triiodothyronine; fT4, free thyroxine; HT, Hashimoto’s thyroiditis; IOL, intraocular lens power; IOP, intraocular pressure; IOPcc, corneal-compensated IOP; IOPg, Goldmann-correlated intraocular pressure; PCA, principal component analysis; RNFL, retinal nerve fibre layer; SFCT, subfoveal choroidal thickness; T3, triiodothyronine; T4, thyroxine; TED, thyroid eye disease; Tg, thyroglobulin; TgAb, thyroglobulin antibodies; TPOAb, thyroid peroxidase antibodies; TSH, thyroid-stimulating hormone.
