# Supplementary material for: Association between Thyroid Function and Ocular Parameters
Source: Biology (Basel). 2022 Dec 18;11(12):1847. doi: 10.3390/biology11121847 (PMC9776046; doi:10.3390/biology11121847)
Supplement: Supplementary file 1 [file biology-11-01847-s001.zip › biology-2035351-supplementary.pdf]

Correlation of optical parameters with TSH, thyroid hormones, Tg, TgAb and TPOAb levels

Before analysis, TSH levels were log-transformed (since TSH distribution was right-skewed, following an approximately log-normal distribution). Several optical parameters correlated with TSH, thyroid hormones, Tg, TgAb and TPOAb levels. However, after the Bonferroni correction for multiple comparisons, only the associations presented in **Table S1** remained significant.

Table S1. Correlation of optical parameters with TSH, thyroid hormones, Tg, TgAb and TPOAb levels.

	TSH	fT4	Tg	TgAb	TPOAb
Spherical power of left eye	$r_S = -0.05$, $p = 0.002$		$r = 0.06$, $p = 0.001$	$r_S = 0.101$, $p < 0.001$	$r_S = 0.081$, $p < 0.001$
Spherical power of right eye			$r = 0.06$, $p < 0.001$	$r_S = 0.009$, $p < 0.001$	$r_S = 0.085$, $p < 0.001$
Cylinder power of left eye	$r_S = -0.05$, $p = 0.002$			$r_S = 0.115$, $p < 0.001$	$r_S = 0.102$, $p < 0.001$
Cylinder power of right eye				$r_S = 0.110$, $p < 0.001$	$r_S = 0.089$, $p < 0.001$
IOL of left eye		$r = -0.05$, $p = 0.002$			
IOL of right eye		$r = -0.067$, $p < 0.001$	$r_S = 0.057$, $p = 0.001$		
Posterior chamber length of right eye		$r = -0.08$, $p = 0.002$ (in men)			
Lens thickness of right eye					$r_S = -0.053$, $p = 0.001$

fT4, free thyroxine; IOL, intraocular lens power; Tg, thyroglobulin; TgAb, thyroglobulin antibodies; TPOAb, thyroid peroxidase antibodies; TSH, thyroid-stimulating hormone. *$p \leq 0.002$.

Table S2. Analysed optical parameters, as well as demographic data and biochemical measurements across the thyroid function groups.

Variable	Euthyroid	Euthyroid with positive antibodies[a]	Subclinical hypothyroid[b]	Clinical hypothyroid[c]	Subclinical and clinical hyperthyroid[d]	p-value
Age<br>[n]	54<br>(42 – 65)<br>[2912]	55<br>(44 – 66)<br>[750]	53<br>(35 – 65)<br>[314]	57<br>(44 – 66)<br>[127]	63<br>(40 - 71)<br>[46]	0.523[a], 0.077[b], 0.210[c], 0.094[d]
TSH (mIU/l)<br>[n]	1.5<br>(1.1 – 2.1)<br>[2918]	1.7<br>(1.2 – 2.4)<br>[752]	4.2<br>(3.9 – 5.3)<br>[314]	5.8<br>(4.2 – 8.5)<br>[127]	0.08<br>(0.03 – 0.19)<br>[47]	<0.001[a], <0.001[b], <0.001[c], <0.001[d]
fT4 (pmol/l)<br>[n]	13.1<br>(12.1 – 14.2)<br>[2918]	13<br>(12.1 – 14.2)<br>[752]	11.9<br>(10.9 – 13.1)<br>[314]	9.9<br>(8.9 – 10.1)<br>[127]	17.1<br>(13.1 – 19.2)<br>[47]	0.194[a], <0.001[b], <0.001[c], <0.001[d]
fT3 (pmol/l)<br>[n]	4.4<br>(4.3 – 4.8)<br>[2918]	4.4<br>(4.2 – 4.8)<br>[752]	4.3<br>(3.9 – 4.7)<br>[311]	3.7<br>(3.2 – 3.9)<br>[127]	5.7<br>(4.5 – 6.2)<br>[46]	0.651[a], <0.001[b], <0.001[c], <0.001[d]
Tg (ng/ml)<br>[n]	10.1<br>(6 – 16.7)<br>[2317]	9.2<br>(3.3 – 16.9)<br>[589]	10.6<br>(5.3 – 16.9)<br>[272]	11.9<br>(7 – 22.1)<br>[97]	11<br>(6.1 – 24.2)<br>[36]	0.145[a], 0.122[b], 0.013[c], 0.130[d]
TgAb (IU/ml)<br>[n]	6.7<br>(5 – 11.5)<br>[2917]	101<br>(17.8 – 157)<br>[751]	12.1<br>(5 – 101)<br>[312]	26.5<br>(7.2 – 211.5)<br>[125]	10.5<br>(5.1 – 110)<br>[47]	<0.001[a], <0.001[b], <0.001[c], 0.002[d]
TPOAb (IU/ml)<br>[n]	2.8<br>(1.3 – 6.5)<br>[2908]	39.3<br>(20.1 – 115)<br>[750]	9.4<br>(2.3 – 82.7)<br>[313]	17.3<br>(3.9 – 153.3)<br>[126]	9.9<br>(1.9 – 37.9)<br>[47]	<0.001[a], <0.001[b], <0.001[c], 0.004[d]
Right eye: posterior chamber length (mm)<br>[n]	15.8<br>(15.2 – 16.5)<br>[2514]	15.8<br>(15.2 – 16.4)<br>[653]	15.8<br>(15.2 – 16.5)<br>[270]	15.5<br>(14.8 – 16.3)<br>[107]	16<br>(15.3 – 16.9)<br>[39]	0.549[a], 0.767[b], 0.023[c], 0.447[d]

Right eye: axial length (mm)<br>[n]	23.1 ± 1.4<br>[2514]	23 ± 1.1<br>[653]	23.2 ± 1.2<br>[270]	22.9 ± 1.1<br>[107]	23.1 ± 1.6<br>[39]	0.097[a], 0.735[b], 0.065[c], 0.832[d]
Right eye: IOL (D)<br>[n]	21.5<br>(19.8 – 23.1)<br>[2483]	21.5<br>(20.2 – 22.9)<br>[648]	21.3<br>(20.3– 22.8)<br>[269]	22.4<br>(20.8 – 24)<br>[106]	21.9<br>(20.1 – 23.9)<br>[38]	0.558[a], 0.201[b], 0.005[c], 0.755[d]
Right eye: corneal thickness (µm)<br>[n]	559.9 ± 36.5<br>[2504]	559.7 ± 35.3<br>[655]	559.3 ± 36.6<br>[269]	558.6 ± 39.1<br>[107]	577.3 ± 36.1<br>[39]	0.917[a], 0.810[b], 0.719[c], 0.003[d]
Right eye: lens thickness (mm)<br>[n]	4.4 ± 0.5<br>[2495]	4.4 ± 0.4<br>[649]	4.4 ± 0.5<br>[267]	4.4 ± 0.4<br>[107]	4.3 ± 0.5<br>[38]	0.150[a], 0.122[b], 0.978[c], 0.127[d]
Right eye: cylinder power (°)<br>[n]	-0.323 ± 0.920<br>[2491]	-0.265 ± 0.885<br>[651]	-0.286 ± 0.865<br>[273]	-0.315 ± 0.913<br>[106]	-0.597 ± 1.262<br>[40]	0.146[a], 0.523[b], 0.928[c], 0.064[d]
Right eye: spherical power (D)<br>[n]	-0.750<br>(-2 – 0.250)<br>[2486]	-0.500<br>(-1.500 – 0.370)<br>[649]	-0.750<br>(-2 – 0.185)<br>[273]	-0.750<br>(-2.250 – 0.563)<br>[106]	-0.750<br>(-1.840 – 0.188)<br>[40]	<0.001[a], 0.434[b], 0.839[c], 0.188[d]
Right eye: anterior chamber angle (°)<br>[n]	87.7 ± 56.9<br>[2140]	87.7 ± 58.7<br>[554]	93.2 ± 54.8<br>[252]	86 ± 56.1<br>[93]	89.8 ± 57.1<br>[33]	0.997[a], 0.146[b], 0.778[c], 0.835[d]
Right eye: anterior chamber depth (mm)<br>[n]	3.0 ± 0.4<br>[2514]	2.9 ± 0.4<br>[653]	2.9 ± 0.4<br>[270]	2.9 ± 0.3<br>[107]	2.8 ± 0.3<br>[39]	0.804[a], 0.775[b], 0.046[c], 0.016[d]
Right eye: corneal radius (mm)<br>[n]	7.8 ± 0.3<br>[1374]	7.8 ± 0.3<br>[397]	7.8 ± 0.3<br>[158]	7.7 ± 0.2<br>[50]	7.7 ± 0.2<br>[15]	0.155[a], 0.608[b], 0.803[c], 0.054[d]
Left eye: posterior chamber length (mm)<br>[n]	15.9 ± 1.2<br>[2503]	15.8 ± 1.0<br>[651]	15.8 ± 1.0<br>[271]	15.7 ± 1.1<br>[107]	15.9 ± 1.5<br>[39]	0.285[a], 0.540[b], 0.079[c], 0.845[d]

Left eye: axial length (mm) [n]	23.1 (22.4 – 23.7) [2508]	23 (22.4 – 23.6) [653]	23 (22.5 – 23.8) [271]	23 (22.3 – 23.7) [107]	23.2 (22.6 – 24.3) [39]	0.418[a], 0.264[b], 0.297[c], 0.9335[d]
Left eye: IOL (D) [n]	21.5 (19.8 – 23.2) [2483]	21.8 (20.2 – 23.1) [650]	21.5 (19.9 – 22.8) [270]	22.3 (20.3 – 23.8) [106]	21.1 (19.5 – 22.9) [37]	0.755[a], 0.704[b], 0.007[c], 0.658[d]
Left eye: corneal thickness (µm) [n]	560 ± 36.2 [2496]	561 ± 36.1 [652]	560 ± 37 [269]	556.8 ± 38.8 [107]	575 ± 41.1 [39]	0.524[a], 0.982[b], 0.371[c], 0.010[d]
Left eye: lens thickness (mm) [n]	4.4 ± 0.4 [2491]	4.3 ± 0.4 [651]	4.3 ± 0.5 [270]	4.4 ± 0.4 [106]	4.3 ± 0.5 [37]	0.404[a], 0.712[b], 0.419[c], 0.648[d]
Left eye: cylinder power (°) [n]	-0.326 ± 1.329 [2486]	-0.220 ± 1.126 [656]	-0.352 ± 1.177 [275]	-0.493 ± 1.753 [107]	-0.381 ± 1.443 [40]	0.040[a], 0.755[b], 0.334[c], 0.795[d]
Left eye: spherical power (D) [n]	-0.750 (-2 – 0.250) [2486]	-0.500 (-1.500 – 0.370) [656]	-0.750 (-2 – 0.120) [275]	-0.500 (-2 – 0.500) [107]	-1.000 (-2.625 – -0.063) [40]	0.001[a], 0.722[b], 0.363[c], 0.155[d]
Left eye: anterior chamber angle (°) [n]	87.7 ± 58.6 [2136]	88.0 ± 59.3 [556]	87.1 ± 58.8 [253]	99.2 ± 57.7 [92]	81.4 ± 60.6 [33]	0.930[a], 0.884[b], 0.066[c], 0.538[d]
Left eye: anterior chamber depth (mm) [n]	3.0 ± 0.5 [2508]	3.0 ± 0.4 [653]	3.0 ± 0.5 [271]	2.9 ± 0.5 [107]	2.9 ± 0.4 [39]	0.483[a], 0.211[b], 0.437[c], 0.490[d]
Left eye: corneal radius (mm) [n]	7.8 (7.6 – 8.0) [1378]	7.8 (7.6 – 7.9) [397]	7.8 (7.6 – 8.0) [160]	7.7 (7.6 – 8.0) [53]	7.7 (7.6 – 7.8) [15]	0.140[a], 0.931[b], 0.431[c], 0.649[d]

Data are presented as Mean ± SD or Median (25-75th percentile). Number of participants is given in the square brackets [n]. P-value denotes the p-value of a t-test for the comparison of each thyroid function group with the euthyroid group. [a]p-value for the Euthyroid with positive antibodies, [b]p-value for the Subclinical hypothyroid, [c]p-value for the Clinical hypothyroid, [d]p-value for the Subclinical and clinical hyperthyroid group. fT3, free triiodothyronine; fT4, free thyroxine; IOL, intraocular lens power; n, number of participants; Tg, thyroglobulin; TgAb, thyroglobulin antibodies; TPOAb, thyroid peroxidase antibodies; TSH, thyroid-stimulating hormone.
